# Use of Agricultural Wastes for the Immobilization of Metals in Polluted Soils in Lagos State, Nigeria

**DOI:** 10.5696/2156-9614-7-13.56

**Published:** 2017-03-29

**Authors:** Aderonke Oluwabukola Oyeyiola, Waheed Adeosun, Idera A. Fabunmi

**Affiliations:** 1 University of Lagos, Nigeria, Yaba, Lagos, Nigeria; 2 Nigerian Institute for Oceanography and Marine Research, Lagos Nigeria

**Keywords:** bioavailability, cow dung, disodium hydrogen phosphate (DSHP), heavy metals, remediation, sawdust

## Abstract

**Background.:**

An increase in human and industrial activities has led to an increase in the pollution of soils by metals. If these metals become bioavailable, there is potential for human exposure, leading to possible health effects. Remediation of soils is important to ensure that soil is suitable for agricultural purposes.

**Objectives.:**

To explore the use of sawdust and cow dung to remediate contaminated soil by reducing the bioavailability of metals.

**Methods.:**

Soil samples were collected from Oke Afa dumpsite and Owode Onirin metallic store and total metal concentrations were determined by digesting with aqua regia and analyzed using flame atomic absorption spectrophotometry. The effect of varied dosages (1–5 g) of cow dung and sawdust on the immobilization of the selected metals was investigated, and results were compared with disodium hydrogen phosphate (DSHP) amendments. A single reagent leaching test was carried out with 0.1 M calcium chloride to determine the bioavailability of metals before and after amendment.

**Results.:**

Before amendment, the amount of bioavailable metal in the soil sample from Oke Afa dumpsite was 57.5 mg/kg, 194 mg/kg and 17.5 mg/kg for copper (Cu), lead (Pb) and zinc (Zn), respectively, while in the second soil sample, from Owode Onirin metallic store, the amount of bioavailable Cu, Pb and Zn was observed to be 19.9 mg/kg, 36.4 mg/kg and 11.1 mg/kg, respectively. Up to 73.4% of mobile Zn was immobilized using DSHP, while cow dung and saw dust were effective in the immobilization of Cu and Pb, but not Zn.

**Conclusions.:**

Cow dung and sawdust reduced the bioavailability of copper and lead, while Zn can be effectively immobilized with the use of DSHP. It was generally observed that the bioavailability of heavy metals decreased with increasing dosage of the amendments.

## Introduction

Soils may become polluted by metals through urban and industrial activities such as mine tailings, disposal of high metal wastes, smelting and refining, e-waste, production of gasoline, paint production and use, land application of fertilizers, wastewater irrigation, coal combustion residues, spillage of petrochemicals and atmospheric depositions.^1^,^2^ These metals are of environmental concern because they are toxic and non-biodegradable. They persist in the environment and may be a threat to both human health and the ecosystem.^3^ Depending on the chemical form (species) in which they occur, they may accumulate in soil and become mobile and bioavailable to plants, animals and humans.^4^ Studies have linked diseases such as cancer to exposure to metals in the environment. Jaffar et al. reported three cases of patients who developed symptoms of skin cancer after chronic exposure to arsenic-contaminated well water in Malaysia.^5^ Studies in an Egyptian population found that serum cadmium levels were higher in 31 new cancer patients compared to control samples.^6^ Again, a positive correlation was observed between the incidences of oral cancer and metal pollution levels in soils in eastern Taiwan.^7^ Human exposure to metals, especially in soils in sub-Saharan Africa, has become a major health risk to humans and animals and remediation of these soils is important.^8^

Soil remediation using efficient, economic and non-destructive techniques can reduce contamination and preserve soil, which is a non-renewable natural resource.^9^ Remediation techniques for contaminated soil involve either immobilization or extractive processes. They include immobilization, landfill, soil flushing and washing, phytoremediation, electro-remediation and excavation.^10^,^11^ Due to the limitations of extractive methods, immobilization techniques, which are more economic, are more widely used for the remediation of metals in soils. Immobilization techniques aim at reducing leachability and hence bioavailability of the contaminants by binding them to suitable binders which can also improve the physical properties of the soil, thus reducing metal toxicity.^12^,^13^

A variety of materials such as carbonates, phosphates, rock phosphates, zeolites, lime, clay minerals, manganese oxide and organic materials have been used as immobilization agents. They have been found to be successful in mitigating soil metal contamination by significantly reducing the bioavailability of metals to plants due to increase in soil pH.^14–16^ Agricultural wastes such as green manure, animal excrement and peat have also been found to be effective.^11^ They have been found to effectively remediate heavy metal contaminated soil by transforming heavy metals from soluble and exchangeable fractions to those associated with organic matter, carbonate fractions and residual fractions, which are unavailable to plants.^10^,^17^

Sawdust and cow dung are agricultural wastes which are cheap, environmentally friendly and available in large quantities in Nigeria. To the best of our knowledge, this paper is the first to present and examine the use of sawdust and cow dung as low cost and environmentally friendly immobilization agents for the remediation of metals such as chromium (Cr), copper (Cu), nickel (Ni), lead (Pb) and zinc (Zn) in soils. The bioavailability of metals before and after remediation was determined by leaching with calcium chloride, and the effectiveness of the immobilization agents was compared with disodium hydrogen phosphate (DSHP). This study characterizes the efficiency of these agricultural wastes as immobilization agents of metals in contaminated soils.

**Abbreviations**
*CaCl_2_*: Calcium chloride
*CEC*: Cation exchange capacity
*Cr*: Chromium
*Cu*: Copper
*DSHP*: Disodium hydrogen phosphate
*Ni*: Nickel
*Pb*: Lead
*Zn*: Zinc

## Methods

### Sample Collection and Properties

The soil samples used for remediation were collected from Oke Afa dumpsite (N06° 31.780, E003° 19.012) and Owode Onirin metallic store which is a hub for metal, steel, and iron sales (N06° 36.480, E003° 24.719) in Lagos State, southwest Nigeria. The samples (four sub samples from each of the locations) were randomly collected (at a distance of about 1 m apart) (0–10 cm deep) and thoroughly mixed (homogenized) together to obtain a composite sample for each of the two locations. Representative samples of each of the soils was homogenized, air dried, sieved through a wire mesh of less than 2 mm and used for the determination of pH, organic matter and pseudo-total metal concentration. The remaining samples were used for the determination of other soil properties and remediation studies. The pH was determined using British Standard ISO 10390.^18^ An aliquot of 5 g soil sample was added to 10 ml of 0.1 mol dm^−3^ calcium chloride (CaCl_2_) solution and agitated for 30 mins. The pH of the supernatant was taken using a pre-calibrated Mettler Toledo pH meter. The organic matter content was determined using the Walkley-Black method of titrating the added dichromate solution with ferrous ammonium sulphate using a ferroin indicator as described by Oyeyiola et al.^19^ The bulk density of the soil was determined by gravimetry, using the method of Miroslav and Vladimir.^20^ This was carried out by weighing 10 g of oven dried sample (105° C) and then compacting in a measuring cylinder. The specific gravity and grain size analysis were performed by granulometry and the Bouyoucos hydrometer method, respectively.^21^,^22^ This was done by first separating the soil into different particle sizes and the proportions of the fractions were determined by sedimentation using a Calgon solution.

### Pseudo-total Metal Determination of Soil Samples and Amendment Agents

Each of the 2 soil samples (1 g) was digested with 20 ml aqua regia on a hot plate for 2 hours.^2^,^23^ The digests were filtered using Fisherbrand QL 100 filter paper (11 cm) into 50 ml standard flasks, made up to the mark with distilled water and stored in plastic bottles at a temperature of 4°C prior to analyses. The soil samples were digested in triplicates. The quantification of the analytes was performed by flame atomic absorption spectrophotometery, using a Perkin Elmer Analyst 200 instrument with air–acetylene flame under optimal conditions. For quality control, the pseudo-total metal analyses were carried out in duplicate and a secondary reference material (GLAURM) was used. GLAURM is an urban soil secondary reference material prepared by participants in the European Union URBSOIL project.^24^ The metal content of the sawdust and cow dung were also determined and blank analysis was carried out.

### Bioavailability Studies

Soil samples (10 g) were weighed in a plastic bottle and 50 ml of 0.1 mol dm^−3^ CaCl_2_ extracting solution was added while maintaining the pH of the soil. The mixture was agitated at 105 rpm for 2 hours in 100 ml sample bottles. The mixture was centrifuged and allowed to settle. The supernatant was decanted through a Whatman filter paper (11 cm) in a 100 ml standard flask, made up to mark with distilled water and then analyzed using flame atomic absorption spectrophotometer.^25^,^26^ The same procedure was also performed for the remediated soil samples.

### Amendments

The amendments used include disodium hydrogen phosphate, sawdust and cow dung. Analytical grade disodium hydrogen phosphate (Na_2_HPO_4_) with a purity of 99.9% was purchased and used for the soil remediation study. The sawdust was obtained from a local sawmill in Lagos State, Nigeria. The sawdust was washed with distilled water three times to ensure that all dirt particles were removed. It was air dried and then oven dried at 60° C for 24 hours. The dried sawdust was then sieved through a 0.6 mesh and kept in a plastic container for remediation analysis. The cow dung used was obtained from a cattle ranch in Lagos State.

For the remediation studies, 100 g each of soil sample was weighed and mixed with varied amounts of amendment (1 g, 2 g, 3 g, 4 g and 5 g), placed into transparent plastic bags (opened) and allowed to stand for 30 days to allow for proper remediation. In order to simulate field conditions, the samples were occasionally wetted with distilled water.

## Results

### Physicochemical Properties of Soil Before Remediation

The physicochemical properties of the soil samples were determined and the results are presented in Table 1.

**Table 1:** Physicochemical Properties of Soil Samples from Owode Onirin Metallic Store and Oke Afa Dumpsite

Parameters	Metallic store soil	Dumpsite soil
pH	7.5	8.6
Bulk density	2.07	0.86
Specific gravity	2.10	0.85
Clay (%)	12.2	43.1
Sand (%)	55.5	28.7
Gravels (%)	15.4	9.80
Organic matter (%)	7.61	9.95
CEC (cmol/kg)	3.81	7.38

Metallic store soil was found to have a neutral pH (7.5) compared with that of dumpsite soil, which was alkaline (8.6). The soil texture analysis indicated that the dumpsite soil sample (43.1) was clayey sandy, while the metallic store soil (55.5) was sandy loamy. Cation exchange capacity (CEC) observed in metallic store soil (3.81) was considerably lower than that of dumpsite soil (7.38), while percentage organic matter was shown to be 7.61 in metallic store soil and 9.95 in dumpsite soil.

### Pseudo-total Metal Concentration in Soil Samples, Reference Materials and Immobilizing Agents

For the determination of the pseudo-total metal concentration in soils (*Table 2*), analysis of the secondary reference material showed an agreement between the results obtained and the target values. The results of the found values were within three standard deviations of the target values, showing good analytical laboratory performance.

**Table 2:** Pseudo-total Metal Concentration in Samples and Reference Materials (mg kg^−1^)

	Cr	Cu	Ni	Pb	Zn
Metallic store soil	138	1020	90.5	206	139
Dumpsite soil	72.5	3810	112	1170	347
Cow dung	ND	1.28	13.4	ND	9.4
Sawdust	ND	ND	ND	ND	2.1
Secondary Reference Material (GLAURM)^24^					
Target	43.2 ± 3.0	111 ± 5	*	387 ± 25	177 ± 11
Found	47.1 ± 4.5	107 ± 12	*	400 ± 18	168 ± 8

**Abbreviations:** *ND, below instrument detection limit*

The concentration of metals in the amendments were observed to be generally low. The below instrument detection limit was −13.4 mg kg^−1^ for cow dung and the below instrument detection limit was −2.1 mg kg^−1^ for saw dust. The concentration of the metals ranged from 90.5–1020 mg kg^−1^ for metallic store soil and 72.5–3810 mg kg^−1^ for dumpsite soil.

### Bioavailability of Metals in Soils Before Remediation

Bioavailability of metals in soils before remediation carried out in both samples (*Table 3*) showed Pb had the highest percentage mobility at 17.67% (metallic store) and 16.58% (dumpsite), where 36.4 mg kg^−1^ of Pb was mobile out of a total of content of 206 mg kg^−1^ in metallic store soil and 194 mg kg^−1^ from a total content of 1170 mg kg^−1^. This was followed by Zn, where 11.1 mg kg^−1^ mobile content was found from a total content of 139 mg kg^−1^ (%mobility 7.99) in metallic store soil and 17.5 mg kg^−1^ mobile content from a total concentration of 347 mg kg^−1^ (%mobility 5.04) in dumpsite soil. Bioavailable Ni in metallic store soil was 2.87 mg kg^−1^ from a total content of 90.5 mg kg^−1^ (%mobility 3.17) and 2.08 mg kg^−1^ from a total content of 211 mg kg^−1^ (%mobility 1.86) in dumpsite soil. Chromium was shown to have a mobility of 2.58%, with a mobile content of 3.56 mg kg^−1^ from a total of 138 in metallic store soil and 1.68% mobility was found in dumpsite soil, with a mobile content of 1.22 mg kg^−1^ from a total content of 72.5. Copper had the lowest mobility in both samples, with 1.95% and 1.51% mobility in metallic store soil and dumpsite soil, respectively. Copper also had the highest total concentration in both samples with 1020 mg kg^−1^ (19.9 mg kg^−1^ mobile content) in metallic store soil and 3810 mg kg^−1^ (57.5 mg kg^−1^ mobile content) in dumpsite soil.

**Table 3:** Bioavailability of Metals in Soils and Amendments Before Remediation

Metals	Cow Dung (mg kg^−1^)	Sawdust (mg kg^−1^)	Dumpsite soil (mg kg^−1^)	Dumpsite soil (%)	Metallic store soil (mg kg^−1^)	Metallic store soil (%)
Cr	ND	ND	1.22	1.68	3.56	2.58
Cu	ND	ND	57.5	1.51	19.9	1.95
Ni	ND	ND	2.08	1.86	2.87	3.17
Pb	ND	ND	194	16.6	36.4	17.7
Zn	1.8	1.2	17.5	5.04	11.1	7.99

Abbreviations: ND, below instrument detection limit

### Immobilization of Metals in Soils Using Different Immobilization Agents

**Figure 1:**
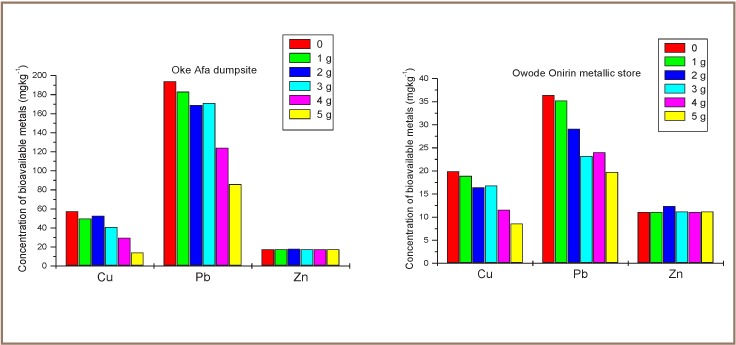
Concentration of bioavailable metals with varied dosages of cow dung

The order of effectiveness of the different dosages of cow dung used for amendment did not follow the same trend for all the metals. Bioavailability of Cu and Pb decreased with increasing amounts of amendments using 1–5 g of cow dung for both types of soils, but at 5 g the concentration of the bioavailable Cu and Pb was still as high as 14.1 mg kg^−1^ and 85.9 mg kg^−1^, respectively, for dumpsite soil and 8.60 mg kg^−1^ and 19.7 mg kg^−1^, respectively, for metallic store soil (*Figure 1*). A similar trend of immobilization of Pb and Cu in metallic store soil was observed in Cr, with optimum immobilization obtained at 4 g (2.25 mg kg^−1^ with 36.8% immobilization) which was higher than for 5 g (2.84 mg kg^−1^ with 20.2% immobilization) while in dumpsite soil, immobilization using 3 g was shown to significantly reduce the concentration of Pb and Cu to 0.31 mg kg^−1^ with 74.6% immobilization. Zinc in both soil samples remained fairly constant, irrespective of the amount of cow dung added.

**Figure 2:**
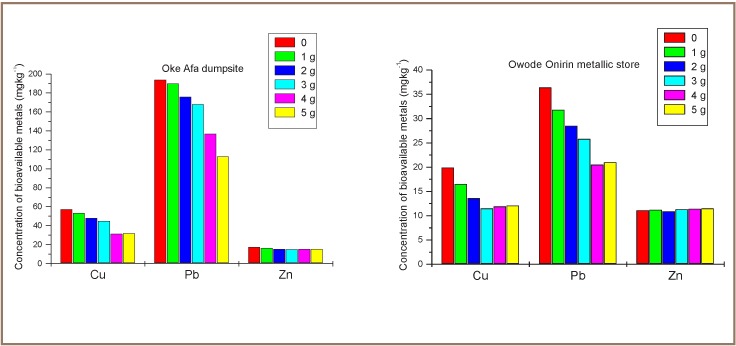
Concentration of bioavailable metals with varied dosages of sawdust

Figure 2 shows the concentrations of the analyzed heavy metals in both types of soil samples before and after treatment with sawdust. Immobilization of Cu, Pb and Zn using sawdust as an amendment was in no particular order, and a decrease in their bioavailability as the amount of amendment used varied from 1 to 4 g. With 4 g of amendment applied, the bioavailability of Cu decreased from the initial concentration of 57.5 mg kg^−1^ to 31.5 mg kg^−1^ in dumpsite soil and with 3 g amendment it decreased from 19.9 mg kg^−1^ to 11.5 mg kg^−1^ in metallic store soil. However, increasing the amendment to 5 g showed a slight increase in the bioavailability of Cu in dumpsite soil. A similar trend was observed for Pb in metallic store soil, while the bioavailability of Pb decreased with increasing dosage of sawdust in dumpsite soil. The optimum dosage for Pb immobilization was seen with 4 g sawdust, reducing the mobile concentration of Pb to 20.5 mg kg^−1^ (43.7% immobility) in metallic store soil, while the optimum dosage of Cu immobilization was with the use of 3 g sawdust, reducing its concentration to 11.5 mg kg^−1^, immobilizing 42% of mobile Cu. However, for dumpsite soil, the optimum dosage for immobilizing Cu was with 4 g sawdust, reducing its mobile content to 31.5 mg kg^−1^ (45.2% immobility), while 5 g dosage was best for effectively immobilizing Pb to a concentration of 113 mg kg^−1^ (41.8% immobility).

**Figure 3:**
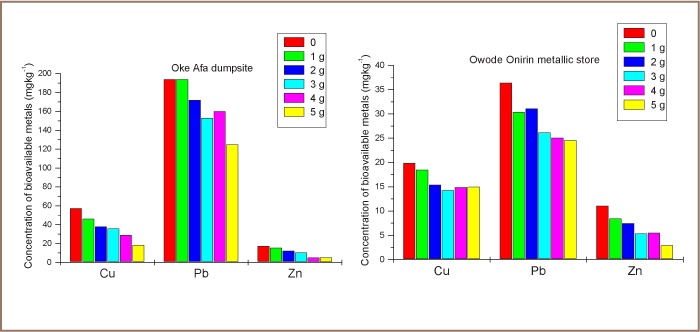
Concentration of bioavailable metals with varied doses of DSHP

The results of immobilization with various dosages of DSHP are shown in Figure 3. In the metallic store soil, the optimum dosage for Cu immobilization to a concentration of 14.3 mg kg^−1^ was found using 3 g phosphate, which immobilized about 28.1% of mobile Cu. It was observed that 5 g phosphate immobilized 32.4% and 73.4% of Pb (24.6 mg kg^−1^) and Zn (2.95 mg kg^−1^), respectively. However, for dumpsite soil, the optimum dosage for immobilizing Cu to 18.4 mg kg^−1^ (68% immobility) and Pb to 125 mg kg^−1^ (35.6% immobility) was with 5 g phosphate, while 4 g phosphate showed a considerable decrease in the mobility of Zn, reducing it from 17.5 mg kg^−1^ to 5.15 mg kg^−1^ at 70.6% immobility.

## Discussion

Soil properties played a major role in the mobility of metals in the soil samples.^16^ Mobility was observed to be higher for all metals in metallic store soil compared to dumpsite soil, suggesting that pH, one of the most important factors in determining the mobility of heavy metals due to its effects on solute concentration and absorption in soil, was an important factor in the results of the present study.^27^ In addition, the clayey nature of dumpsite soil gives it the potential to bind strongly to heavy metals, as clays are known for their ability to effectively remove heavy metals by specific adsorption and cation exchange.^28^ Percentage organic matter has been shown to increase sorption capacity for metal ions to soil particles. Kabata-Pendias showed that Cu, Zn, Pb and Cd are absorbed on organic matter which generates stable forms and lead to their accumulation in the organic horizon of soil.^29^ The higher organic matter in dumpsite soil, which reduced mobility, could be attributed to the decomposition by microorganisms of various household and industrial wastes being deposited at the dumpsite. However, minimal biological activities occur at the metallic store site where samples were collected. The CEC value was found to be higher in dumpsite soil compared to metallic store soil, and this could be a result of higher organic matter content of the soil. Heba and Mikhail indicated that soils with higher levels of clay and organic matter have a higher sorption capacity.^28^ This explains the higher percentage mobility of heavy metals shown in metallic store soil compared to dumpsite soil.^30^ The properties of the two soil samples appear to be slightly different from each other, therefore these samples may represent different types of soils.

As a result of lower pH, percentage clay, CEC and organic matter, mobility of heavy metals was significantly higher in metallic store soil. Lead was found to be more mobile compared to the other heavy metals studied. Mobility of Pb could be a result of other factors not considered in this study such as sulphate reduction through formation of sulphide alloids. Another possible explanation is the decrease in sorption of Pb in the presence of complexing ligands and formation of such complexes may greatly increase the mobility of lead in soil.^31^ Contrary to Pb, Cu exhibited a lower mobility potential, and this could be attributed to its ability to form strong complexes in soil and also absorb to a great extent to soil and soil constituent.^32^ The concentration of bioavailable Cu and Pb as assessed with CaCl_2_ in dumpsite soil was observed to be high, indicating that these amounts may be taken up by plants and migrate into the food chain, with the potential to cause undesirable effects in the human body. The mobile content of Cu, Pb and Zn in dumpsite soil was shown to be more bioavailable than metallic store soil, and this may be a result of their much higher total concentration in dumpsite soil compared to the total concentration in metallic store soil.

Generally, the levels of metals in both soils were high and therefore they can be said to be polluted. The concentrations of all of the studied metals were much higher than levels of metals in uncontaminated soils or background concentration of metals in soils, which were 37, 17, 13, 16 and 48 mg kg^−1^ for Cr, Cu, Ni, Pb and Zn, respectively.^33^ Although the amount of bioavailable metal was low compared to the total concentration, it still indicates the need for immobilization of the metals in soils so that they do not migrate into the food chain. Since Cu, Pb and Zn were the most mobile, immobilization of these metals was investigated.

The introduction of cow dung at dosages ranging from 1 g to 5 g was shown to significantly reduce the mobility of the studied heavy metals with the exception of Ni in both samples. This can be explained by its ability to introduce additional binding sites for the heavy metals, making them less mobile and available.^34^ Poor immobilization of Ni was also shown in a previous study, which found that the distribution coefficients of Cd and Ni were low after treatment with cow dung, indicating their bioavailability.^35^ In addition, the poor immobilization of Ni in the soil samples could be as a result of the presence of Ni in cow dung before remediation. Sawdust was shown to reduce the mobility of Ni and Cr compared to cow dung and DSHP, however, immobility of Zn was not affected by the addition of sawdust in both samples, possibly due to its initial presence in the sawdust before amendment.

Using varying masses of DSHP for immobilization of metals in soils, it was observed that the concentrations of the analyzed metals in both types of soil samples before treatment reduced with varying dosages of DSHP. In most cases, the concentrations of available Cu, Pb and Zn in the soil for both samples decreased consistently with increasing dosage of DSHP, suggesting that DSHP reduced the bioavailability of these heavy metals. The result obtained is in agreement with a study on heavy metal immobilization using diammonium phosphate, where diammonium phosphate was used for immobilizing Cd, Pb and Zn eluted from the contaminated soil.^36^ In a related study, calcium magnesium phosphate decreased the Cu and Cd concentration in soil by 19.4% and 9.4%, respectively, which is in agreement with the present study, although a higher decrease was found for DSHP.^37^ Chemical immobilization research using phosphate addition has included mineral apatite and synthetic hydroxyapatite materials. These materials have proven to be effective at reducing the solubility and bioavailability of heavy metals through the formation of metal-phosphate minerals.^14^,^25^,^38^

**Figure 4:**
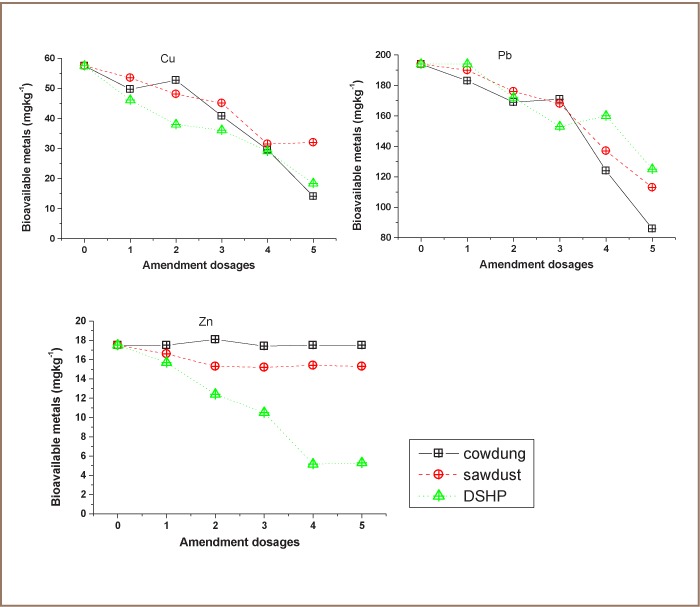
Comparison of the effect of amendments on soil from Oke Afa dumpsite

**Figure 5:**
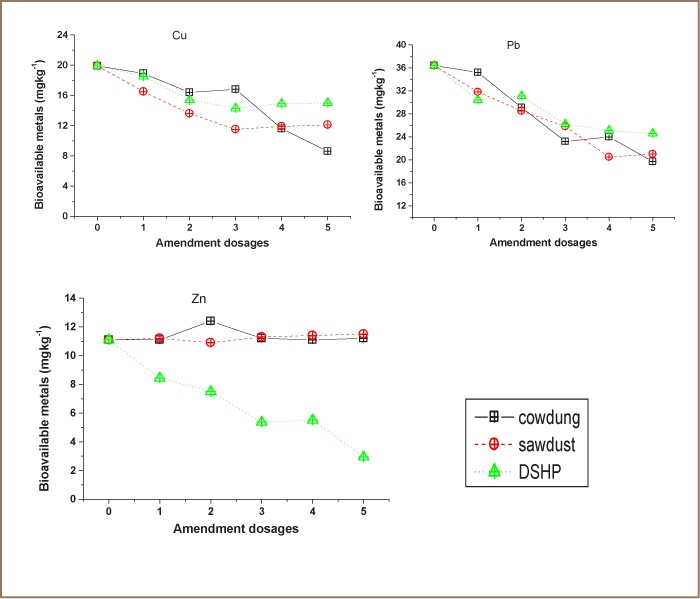
Comparison of the effect of amendments on soil from Owode Onirin metallic store

A comparison of the three amendments revealed that the best expressed dosages of cow dung and sawdust had lower immobilization for Zn compared to DSHP in both samples. Cow dung and saw dust had no effect on the immobilization of Zn. This is in agreement with Kubna et al., who showed that compost amendment enhanced the bioavailability of Zn.^39^ Cow dung had the best immobility for Cu and Pb in dumpsite soil (*Figure 4*), and Cu in metallic store soil (*Figure 5*). Cow dung and sawdust exhibited the same amendment capability in dumpsite soil. The strong affinity of sawdust to immobilize heavy metals agrees closely with previous studies.^40^ It was generally observed that cow dung and sawdust showed greater immobilization in dumpsite soil which could be due to the soil properties, with the exception of Ni for cow dung and Pb for sawdust. The general trend of decrease in mobility with an increase in adsorbent dose has been reported for metal adsorption by oak sawdust.^40^ Statistical analysis using the t test showed that there was no significant difference (p > 0.05) in the use of the three amendments for immobilizing these metals.

## Conclusions

Remediation of heavy metal contaminated sites has become necessary due to the harmful effect of these heavy metals. The present study investigated the effect of cow dung, sawdust and DSHP on heavy metal bioavailability in soil. Cow dung, sawdust and DSHP appreciably decreased the mobility of copper and lead. Cow dung and sawdust were also effective in reducing the mobility of chromium. The mobility of zinc was only affected by an increase in the dosage of DSHP. Sawdust and DSHP slightly reduced the mobility of Ni, while cow dung showed little or no effect. Therefore, cow dung and sawdust can be used to reduce the bioavailability of copper, lead and chromium, while Zn can be effectively immobilized with the use of DSHP. Statistically, no significant difference was observed in the use of the three amendments, indicating that natural and ecofriendly immobilization agents can be used on a large scale for the remediation of contaminated soils and to ensure that soil is suitable for agricultural purposes.
